# Stress and Dyadic Coping in Personal Projects of Couples – A Pattern-Oriented Analysis

**DOI:** 10.3389/fpsyg.2019.00400

**Published:** 2019-02-28

**Authors:** Tamás Martos, Viola Sallay, Marianna Nagy, Henrietta Gregus, Orsolya Filep

**Affiliations:** ^1^Institute of Psychology, University of Szeged, Szeged, Hungary; ^2^Doctoral School, Semmelweis University, Budapest, Hungary

**Keywords:** stress, dyadic coping, self-regulation, Dyadic Coping Inventory, personal project assessment, dyadic data, cluster analysis, relationship satisfaction

## Abstract

Relational accounts of goal striving have barely considered dyadic coping as an element of the process, nor has dyadic coping research utilized the unique advantages of the goal construct (e.g., in form of personal project assessment) so far. Therefore, the primary aim of the present study was to explore stress and dyadic coping experiences associated with the personal projects of partners in a close relationship. Moreover, we approached data analysis in a pattern-oriented way, instead of using variable-centered linear models. We used cross-sectional data from 270 married and cohabiting Hungarian heterosexual couples (mean age 40.1 ± 11.2 and 37.8 ± 10.9 years for male and female partners, respectively). Partners individually completed an adapted version of the Personal Project Assessment procedure. First, they named an important but stressful personal project. Respondents appraised their experiences with the chosen personal project along several predefined aspects. These included: (1) stress experiences; (2) dyadic coping, using the adapted Dyadic Coping Inventory; (3) positive emotions; and (4) sense of community. The Relationship Assessment Scale was also assessed. Cluster analysis of both partners’ stress experiences, positive and negative dyadic coping strategies in their own personal projects revealed six relationship-level clusters. Cluster solutions represented typical variations of the stress and dyadic coping patterns of the couples, and could be arranged in a three- (lower, medium, and higher stress) by-two (positively vs. negatively balanced dyadic coping pattern) array. Further analyses indicated the general trend that couples with lower (vs. higher) stress together with more positively (vs. negatively) balanced dyadic coping may have experienced better functioning in projects (more positive emotions and higher sense of community) and higher relationship satisfaction. Results confirm that the partners’ pursuit of their personal projects is embedded in their relationship, and their functioning in these projects may partly depend on dyadic coping with the stress that arises during the accomplishment of the project. By using a pattern-oriented approach to dyadic data, we were able to distill stress and coping patterns that capture the specific types of couples’ relationships and indicate the non-linear and multidimensional nature of stress and dyadic coping processes.

## Introduction

People often pursue important personal goals in their lives that are related to the goals of important others. For example, when a husband seeks to arrange a vacation together with his spouse, but the wife has to take an important exam, they will have to cooperate in their personal pursuits in order to maintain a well-functioning relationship. Moreover, the accomplishment of these goals is often accompanied by the experience of stress (c.f., Carver et al., 2008) that requires joint stress management efforts in the relationship. Building on this, in the present study, we aim to connect two domains of relationship functioning in close relationships. First, we focus on systemic accounts of self-regulation: on everyday personal projects of partners as these are embedded in their close relationship; and second, on dyadic coping (and more specifically, on the Systemic Transactional Model) to coping with stress. Then, we link these domains, and describe how a personal-project-based approach may add to our present knowledge about the processes of dyadic coping with stress.

### Personal Goals, Personal Projects, and Close Relationships

The pursuit and successful accomplishment of personal goals are vital parts of individual self-regulation and well-being (e.g., Brunstein, 1993; Baumeister et al., 2007; Klug and Maier, 2015). Recent theoretical approaches to self-regulation have emphasized the fundamentally relational nature of the goal-striving processes. Individual self-regulation is closely interwoven with the self-regulation efforts of important others in the sense that, from a systemic point of view, close relationships themselves may be treated as units of analysis (Fitzsimons and Finkel, 2015; Fitzsimons et al., 2015), whereby individual and relational regulations are circularly related to each other (Finkel and Fitzsimons, 2011; Fitzsimons and Finkel, 2011). While working on their personal goals, individuals are constantly faced with challenges that result from the goals and actions of others. In fact, according to Transactive Goal Dynamics Theory (Fitzsimons and Finkel, 2015; Fitzsimons et al., 2015), personal goals of individuals in a committed relationship may also be regarded as interrelated and linked goals that can be best understood in the context of the close relationship.

Goal constructs have successfully been applied in studies of relationship functioning in general (Kaplan and Maddux, 2002), of relational-level adjustment to life transitions (Salmela-Aro et al., 2010) and of the management of relationship conflicts (Gere and Schimmack, 2013). It was also found that mutual support for partners’ personal goals and strivings significantly contributes toward relationship satisfaction; in return, the experience of a higher relationship quality fosters further support and goal coherence (Molden et al., 2009; Overall et al., 2010; Hofmann et al., 2015).

In these studies, similarly to the individual level research tradition of goal-directed self-regulation, personal goals have often been conceptualized in more concrete terms such as the pursuit of personal strivings, personal projects or actual concerns (c.f., Emmons, 1997). For the present study, we will apply personal projects as the core theoretical and methodological construct. Personal projects are sets of personally important pursuits of individuals that are embedded in their everyday ecological contexts (Little, 1983, 2006). While overarching goals like ‘performing well’ may represent general rules of self-regulation, the project ‘I will pass my next professional exam’ is deeply embedded in the actual context of the person. Moreover, it refers to desired future states as well (e.g., professional achievement). This way, an investigation of personal projects is capable of capturing both the actual social ecological context of an individual’s life and its future-oriented component (Little, 2015). Since close relationships are among the most important contexts of individual goal striving (c.f., Fitzsimons and Finkel, 2015), it may be inferred that personal projects are ideal units for psychological assessment and analysis of relationship related experiences.

### Stress and Coping in Personal Projects

Processes of coping with stress and goal-directed behavior are often treated as distinct concepts. In the stress literature, people often (and implicitly) are depicted as if they were required to face difficulties and stressful circumstances independently of their previous actions. Their agency only appears in their coping efforts, and the quality of coping with these situations influences their quality of life, as well as their health and well-being (Thoits, 2010; Carnes, 2017; Praharso et al., 2017). However, important theoretical work connects stress and coping with self-regulation. For example, Carver et al. (2008) argue that stress and coping can be better understood as linked to goal-directed action. Stress is viewed as goal-related frustration, whereas coping is regarded as the self-regulation process that helps the individual find a new way to accomplish the goal or to disengage from it. When pursuing an ‘exam’ project, for example, one can easily run into difficulties with time management or a complicated learning task. Personal projects themselves may evoke stress in the individual, and coping efforts to regulate this stress become part of project accomplishment. In a similar way, we may assume that partners also have to regulate their personal projects with regard to their relationships, and thus, project-related coping processes may also have relational implications.

### Dyadic Coping With Stress in Close Relationships

Recent theoretical developments concerning stress and coping acknowledge that individual stress often becomes dyadic stress that impacts both members of a couple. According to Bodenmann (1997), dyadic coping involves the joint process of a couple responding to an individual stressor of one of the partners’. The reason for that is to restore the homeostasis of the relationship. In dyadic coping both partners’ stress management skills are activated. The importance of effective dyadic coping is emphasized by the fact that chronic stress can negatively affect communication among partners, as well as the quality and the development of a relationship (Papp and Witt, 2010). In addition, unmitigated stress can increase the chance of divorce in the long term (Bodenmann, 2000, 2005; Story and Bradbury, 2004). Consequently, the recognition, that coping processes have dyadic aspects as well has led to the concept of dyadic coping.

Dyadic stress, dyadic coping and the connection between the two concepts have become an area for extensive research in the last few decades (Bodenmann, 1997; Falconier et al., 2016; Sim et al., 2017; Staff et al., 2017). One of the most often used dyadic coping models is the Systemic Transactional Model (STM, Bodenmann, 1995). STM identifies the circular processes of dyadic coping that involve both partners’ coordinated actions of stress communication, partner’s reactions, and the appraisal of these reactions by the stressed partner. STM also considers common coping: when partners are involved in joint action to handle stress. According to the model, the mutual dyadic coping efforts of partners can be classified as positive (supportive and delegated acts of dyadic coping) or negative (hostile, ambivalent and superficial ways of dyadic coping). Several studies have used STM to investigate the significance of dyadic coping in couples’ lives and its interrelation with relationship satisfaction (c.f., Falconier et al., 2015a, 2016). Positive dyadic coping with stress is associated with well-being and better relationship quality, while negative dyadic coping more often occurs between couples who experience distress in their closeness (Bodenmann et al., 2004; Herzberg, 2013; Falconier et al., 2015a).

By now, the stress-buffering potential of positive dyadic coping skill has been well documented (Merz et al., 2014; Falconier et al., 2015b; Breitenstein et al., 2018; Hilpert et al., 2018), showing that extra-dyadic or external stress (emerging outside of the relationship) may have a spillover effect on intra-dyadic or internal stress (conflict and tension between partners) only when partners’ dyadic coping behaviors tend more toward negative strategies. The adverse effects of unmitigated dyadic stress reinforce STM’s claims about the importance of adequate dyadic coping in long-term relationship functioning. However, further research is needed to understand how processes of stress and dyadic coping are nested in the everyday life context of couples (c.f., Bodenmann et al., 2016) as they construct their life circumstances through their active pursuit of goals, desires and accomplishments. For example, a couple’s stress stemming from one partner’s project (e.g., the wife’s professional exam) can be co-regulated by the couple’s dyadic coping strategies; which, in turn, will impact the accomplishment of the project. Thus, we turn to examining the interrelations of dyadic coping and the relationship-level regulation of personal projects.

### Dyadic Coping, Goals, and Personal Projects

To the best of our knowledge, relational accounts of goal striving have barely considered dyadic coping as an element of the process. Moreover, while in STM goals play an important role in the dyadic coping process on a theoretical level (c.f., Bodenmann, 1995; Bodenmann et al., 2016), dyadic coping research rarely addressed the specific role of goals so far, except for a few but notable examples. For example, Kuster et al. (2017) tested approach and avoidance orientations in relationship goals in relation to dyadic coping strategies. Their results showed that approach-oriented goal striving in romantic relationships was associated with better relational outcomes, including more effective stress communication and better dyadic coping, whereas using avoidance goals in the relationship produced more negative consequences. In another study (Koranyi et al., 2017) researchers found that experiencing partner’s stress might increase implicit preferences for communal goals that, in turn, predicted stronger inclinations to provide support in form of supportive dyadic coping. However, these associations were primarily true for participants with high relationship satisfaction. These studies provide support for the theoretical notion of STM where goals are results of primary and secondary appraisals as well as antecedents of the actual coping behavior (Bodenmann et al., 2016). This way, dyadic coping behaviors may serve specific individual and relationship oriented goals in a stressful situation. For example, supportive dyadic coping helps to fulfill relationship goals by reducing partner’s emotional stress arousal and bad mood while assisting the partner’s own efforts (c.f., Bodenmann et al., 2016, p. 13).

Using a personalized approach to goal striving, Martos et al. (2019) recently extended the personal project assessment methodology to the study of dyadic coping strategies, and demonstrated the reliability and validity of this approach in a pilot study. They argued that the construct of dyadic coping, as formulated in STM, might be included in models of relationship-level regulation of personal projects. Moreover, the assessment procedure might provide a more contextualized methodological approach to dyadic coping as well. With a sample of couples, the authors used the Dyadic Coping Inventory (DCI) to assess dyadic coping strategies. Partners in a dyad were asked to separately choose an actual stressful personal project of their own and to rate this project along the slightly modified items of the DCI. Thus, the procedure tapped into the everyday relationship experiences of the partners, and also assessed dyadic coping behaviors in this context. However, personal projects represent a distinct aspect of the motivational system that is complementary to the goals described in STM. Personal projects are larger scale pursuits in time that may include several forms of relationship regulation processes, among them, dyadic coping; while during the process of dyadic coping partners activate more proximal, actual goals that drive their coping with stress. Thus, personal projects and goals in dyadic coping represent different levels in the complex organization of individual and relational self-regulation.

### The Present Study

Building on and extending the work of Martos et al. (2019), in the present research we connect the following aspects of individual and relationship functioning. We examine individually pursued personal projects of each member of a couple while we assume that their projects and, consequently, their project related experiences are embedded in the broader context of the couple’s relationship. Therefore, we seek to study the partners’ experiences of stress and dyadic coping in the context of their personal projects. The stress experienced by either partner during the accomplishment of their personal project may be approached with dyadic coping strategies jointly applied by the partners. Furthermore, the quality of this stress and dyadic coping process may have an impact on other personal project related experiences and may be associated with relationship functioning in general as well. Successful dyadic coping with project related stress may help to maintain positive emotions in the partners during the accomplishment of the personal project; it may also enhance their sense of community concerning the project (c.f., Fowers and Owenz, 2010; Randall et al., 2013). Consequently, in the context of the personal projects of the partners, we examined the association of stress and dyadic coping with two kinds of experience: positive emotions associated with the personal project and sense of community with the partner in the project. Hereby, positive emotions refer to primarily individual experiences of the partners during the accomplishment of the projects, whereas project related sense of community refers to primarily relational experiences of the partners. Moreover, in the broader context of the relationship, personal project related stress and dyadic coping experiences may be connected to relationship functioning in general as well. Accordingly, we assessed relationship satisfaction, a commonly used indicator of the general functionality of a romantic relationship in relation to stress (Randall and Bodenmann, 2017).

In order to address the holistic, and potentially non-linear nature of this systemic stress and dyadic coping functioning in dyads, we firstly apply a pattern-oriented analytic approach (Bergman and Trost, 2006) to data analysis and explore the relationship level patterns (c.f., Seiffge-Krenke and Burk, 2013; Czikmantori et al., 2018) of stress and dyadic coping experiences in the context of the personal projects of the partners. In the next step, we relate these emergent patterns to a series of outcomes that represent individual and relational functioning, both in the context of the personal projects themselves and in the broader context of the couple’s relationship. Table 1 presents an overview on the conceptual and methodological network of our study. Moreover, we summarize the core aspects and assumptions of our study in detail in the following sections.

**Table 1:** Conceptual and methodological network of the study.

General context	Studied context	Measured experience		Primary reference of the experience	Analytic approach
Couple’s relationship functioning	Partners’ personal projects	Stress		Individual	Pattern-oriented/explorative (Step 1)
		Positive	Dyadic coping	Relational	
		Negative		Relational	
		Positive emotions		Individual	Outcome-oriented/deductive (Step 2)
		Sense of community		Relational	
	Relationship satisfaction in general			Relational	


#### Assessment of the Personal Projects

Treating personal projects as conceptual units (c.f., Little, 2006) provides a powerful way to study personal and interpersonal processes in their everyday context. Moreover, as we have argued previously, dyadic coping with stress may be an important component of the effective relationship-level regulation of personal project attainment. The corresponding methodology of personal project assessment is a flexible and complex measurement tool that is suitable for assessing ecologically valid, contextually embedded experiences of respondents. The typical assessment procedure includes an individual elicitation of personal projects (e.g., “arranging a vacation trip for my parents”), followed by the measurement of several project-related characteristics (Little and Gee, 2007). In our study, we have adapted this personal project based procedure to capture stress experiences and dyadic coping strategies, as well as experiences of positive emotions and sense of community with the partner.

#### Stress and Dyadic Coping in Personal Projects

We used personal project related stress appraisals and dyadic coping strategies as the core units for the pattern-oriented analytic approach. In a previous piece of work (c.f., Martos et al., 2019) we measured only dyadic coping strategies via the personal project assessment procedure, that is, relational experiences in the project. However, since previous research has confirmed that the extent of perceived stress in a relationship may vary considerably, and this can have an adverse effect on relationship functioning (e.g., Merz et al., 2014; Hilpert et al., 2018), the inclusion of personal project related stress appears necessary for understanding the complex relationship between stress and dyadic coping in the partners’ personal project pursuits. Therefore, in addition to a personal project based assessment of dyadic coping strategies, we also aimed to measure the amount of stress that was experienced by the partners during the accomplishment of their individual projects.

#### Pattern-Oriented Analysis

Scientific attention is being increasingly focused on the theoretical and practical differences between two branches of data-analysis strategies, namely the variable- and the pattern-oriented approaches, the latter of which is also called the person-oriented or person-centered approach (for a recent review, see Howard and Hoffman, 2017). Traditional variable-oriented approaches focus on separate individual characteristics (variables) and the linear associations between them. This way, results from a variable-oriented approach inform central tendencies, general rules and grand means. In contrast, a pattern-oriented approach seeks to maintain a holistic view of the individual (Bergman and Trost, 2006). It utilizes a complex set of multiple interdependent characteristics simultaneously and investigates how these characteristics relate to each other in specific ways (i.e., how they form types) and how they interact with each other as parts of a complex integrated system (Bergman et al., 2003; Bergman and Lundh, 2015). Pattern-oriented approach focuses on the whole system as the unit of analysis; in our case, on the couple, and more specifically, on the partners’ interrelated, systemic functioning in their personal project pursuits. This way, the pattern-oriented approach can be viewed as complementary to the variable-oriented approach (Asendorpf, 2003; Czikmantori et al., 2018).

While research into dyadic relationships has been dominated by variable-oriented studies – for example, with the extensive use of the Actor-Partner Interdependence Model (APIM; Kenny and Ledermann, 2010) –, studies with a pattern-oriented approach to dyadic data are also attracting scholarly attention (Gagliardi et al., 2013; Seiffge-Krenke and Burk, 2013; Cao et al., 2015; Wood et al., 2015; Czikmantori et al., 2018). Accordingly, we assumed that a pattern-oriented approach may be an appropriate way to approach the personal project-related stress and dyadic coping experiences of partners in a close relationship. Since both stress and dyadic coping were assessed in relation to the same concrete personal projects, these experiences describe elements of the integrated, systemic functioning of the couple. Moreover, pattern-oriented data analysis is suitable for revealing complex interaction patterns in multiple characteristics, without the limitations of traditional (variable-centered) two- or three-way interaction analyses. As a result, we expected to explore and identify meaningfully different patterns of stress and dyadic coping in the personal-project-related experiences of couples. We also assumed that these emergent patterns would represent characteristic variations in couples’ relationship functioning.

#### General Assumptions on Outcomes

In addition to the previous explorative analytic step, we planned a deductive, outcome-oriented analysis where the emergent stress and dyadic coping patterns would be compared to a series of potential outcomes of the stress and dyadic coping process. Although pattern-oriented approaches serve for exploratory purposes rather than hypothesis testing (Bergman and El-Khouri, 2003), we may still formulate a couple of general assumptions regarding the associations with outcomes. As a general pattern, we expected that higher stress together with less positive strategies of dyadic coping in personal projects would be associated with a lower level of beneficial experiences in the personal projects themselves (positive emotions and sense of community) as well as lower relationship satisfaction in general. However, the exact configurations of these associations required detailed exploration.

## Materials and Methods

### Procedure

We conducted a cross-sectional study with voluntary participants who filled in an internet-based questionnaire. The respondents were recruited by trained students via an online advertisement on personal forums and social media. The participants were informed about the general aims of the study (i.e., the study of personal goals in a relationship context) and about the anonymity and confidentiality of data handling. Participants explicitly gave their informed consent through responding to the first question. Acceptance of this informed consent question was a prerequisite for subsequently filling in the questionnaire. Participants received no credit or monetary compensation for their participation. The research design was approved by the Unified Ethical Committee for Psychological Research of Hungarian Universities (EPKEB).

### Sample

Table 2 presents descriptive statistics for the participants. In sum, a community sample of 270 married and cohabiting Hungarian heterosexual couples (mean age 40.1 ± 11.2 and 37.8 ± 10.9 years for male and female partners, respectively) were assessed. The average length of relationship was 13.42 years (*SD* = 11.51). Data was missing for 129 couples concerning their number of children; from the remaining 141 couples 49 were raising at least one underage child, while 92 had no children. Respondents rated their subjective financial status as slightly above average (on a 1–10 scale *M* = 6.04). Approximately 28.3% of the participants (92 men and 61 women) had a primary, 58.0% a secondary (147 men and 166 women) and 13.7% (31 men and 43 women) a tertiary level of education.

**Table 2:** Sociodemographic characteristics of the sample.

		Cohabiting					Married				
		
		*N*	*m*	*SD*	Min	Max	*N*	*m*	*SD*	Min	Max
Age	*M*	103	34.07	9.72	23	66	167	43.85	10.45	26	76
	*F*	103	32.54	9.29	21	63	167	40.96	10.65	25	73
Subjective financial status	*M*	102	6.14	1.77	1	10	165	6.07	1.89	1	10
	*F*	103	5.81	1.69	1	9	166	6.09	1.86	1	10
Length of relationship	*M*	94	7.26	7.53	1	45	118	18.33	11.80	1	50
	*F*	93	6.70	5.88	1	35	114	18.14	11.70	1	50
Number of children	*M*	53	0.13	0.52	0	3	87	0.86	1.02	0	4
	*F*	53	0.02	0.14	0	1	87	0.99	1.04	0	4


### Measures

#### Personal Project Assessment

##### Overview

Partners individually completed an adapted version of the Personal Project Assessment procedure. We assessed the experiences of participants related to stressful personal projects via an adapted version of the personal project assessment procedure (see Little and Gee, 2007). As a first step (project elicitation), participants were asked to write a short list of their current personal projects. We defined personal projects as “the goals and strivings that you are currently working on in your everyday life” (see Table 3 for examples of the personal projects in the study). In the second step, respondents selected the “most stressful” personal project from the list, leaving the respondents to define how they interpreted stress in their projects. Finally, participants were instructed to assess their personal experiences with their stressful personal project along several predefined aspects. These included (1) stressful and negative feelings as experienced during working on the projects; (2) project-related dyadic coping actions and evaluations – using the slightly modified items of the Dyadic Coping Inventory; and (3) further project-relevant experiences (i.e., positive emotions and sense of community).

**Table 3:** Examples of couples’ personal projects.

Couple no.	Male partner’s project	Female partner’s project	Coded as
7	Insulate the house	Install insulation in the rooms in the attic	‘Same project’
63	Quit smoking because we will have a baby	Stop smoking due to pending birth of child	‘Same project’
128	Learn photography properly	Improve my English	‘Different projects’
140	Take a nice vacation together	Pass professional exam	‘Different projects’
161	Take less medication	Settle our debts	‘Different projects’
188	Buy a new car	Finish the paintwork in the flat	‘Different projects’


*Coding process referred whether the partners named essentially the same vs. different personal project.*

##### Stress in personal projects

In order to estimate the extent of aggregated stress perceived in the chosen personal project we asked four questions. Two questions concerned the respondent’s own experiences with the project (“How difficult is it for you to work on this project?” and “How much negative emotion/how much tension do you feel while working on this project?”). Two other questions asked about the same issues but from the point of view of the partner; that is, how the respondent perceives the experiences of their partner concerning the project (“How difficult is it for your partner to cooperate in this project?” and “How much negative emotion/how much tension does your partner feel concerning this project?”). Responses were scored on a 7-point, Likert-type scale (1 = low agreement, 7 = high agreement). Averaged scores of own and partner’s stress appraisals were calculated for further analysis for each respondent.

##### Dyadic coping in personal projects

We assessed dyadic coping experiences as they were related to the selected stressful personal projects. For the purposes of this study, we adapted the items of the Dyadic Coping Inventory (DCI, Bodenmann, 2008; Hungarian version: Martos et al., 2012). The same procedure was used as in research by Martos et al. (2019). The 37 items of the DCI assessed the couples’ dyadic coping strategies. Subscales included stress communication (for example “When I feel stressed, I tell my partner openly how I feel, and that I would appreciate his/her support”), supportive, delegated and negative coping (for example “My partner blames me for not coping well enough with stress.”). Respondents were also asked to indicate the stress communication of their partners and the way they react to their partners’ stress in a supportive, delegated, or negative way. Finally, items assessed the frequency of common coping (for example, “We try to cope with problems together, and search for ascertained solutions.”). Two additional items referred to the evaluation of the dyadic coping process (for example, “I am satisfied with the support I receive from my partner, and the way we deal with stress together.”). We used a re-worded version of the DCI in which phrases concerning the items had been modified from general present tense to past tense (e.g., “When I felt stressed I told my partner…” instead of “When I feel stressed I tell my partner…”). Moreover, in the instructions we referred explicitly to the chosen personal project, asking the respondents to indicate how often they experienced the presented coping behaviors in relation to their project in the past few weeks (1 = very rarely, 5 = very often). For further analysis, we calculated summed scores of positive (stress communication, supportive, delegated and common) as well as negative dyadic coping strategies for each respondent.

##### Positive emotions in personal project

In addition to the stress and dyadic coping assessment, respondents were also asked to rate their personal project along further dimensions. Firstly, they rated the amount of positive emotions experienced throughout the project. In addition, participants also rated this aspect from their partner’s point of view; that is, the partner’s positive emotions in the personal project of the respondent. Finally, respondents rated the amount of “joyful experiences together” related to the personal project. Responses were scored on a 7-point, Likert-type scale (1 = low agreement, 7 = high agreement). Ratings of positive emotional experiences (self and attributed to the partner) as well as joyful experiences together were averaged into the “positive emotions in the personal project” variable (henceforth “positive emotions”) for each respondent.

##### Sense of community in personal project

In a similar way, respondents were asked to indicate the extent they regarded the selected project as a “joint project,” (i.e., how much they felt community with their partner concerning the project). Respondents also indicated the extent of agreement to which their partner regarded the project of the respondent as a “joint project.” Responses were scored on a 7-point, Likert-type scale (1 = low agreement, 7 = high agreement). Self and attributed ratings of “joint project” of the personal project were averaged into the “sense of community in the personal project” variable (henceforth “sense of community”).

#### Relationship Assessment Scale

Participants indicated their relationship satisfaction by scoring items using the Relationship Assessment Scale (RAS; Hendrick, 1988; Martos et al., 2014). The seven RAS items assessed the respondent’s satisfaction with their own relationship (sample item: “How well does your partner meet your needs?”). Responses indicate the degree of agreement on a 5-point, Likert-type scale (1 = little agreement, 5 = high agreement). The alpha coefficient indicated excellent internal consistency in the sample (alphas = 0.875 and 0.859 for male and female partners, respectively).

## Results

### Overview of the Analytical Process

After initial data screening, the variables for the study were identified and their basic psychometric properties and bivariate associations were computed (see Table 4 for an overview). As a first analytic step, explorative cluster analysis using the pattern recognition module of the ROPstat software (Vargha, 2016) was run on the aggregated stress and dyadic coping scores of the partners. Since we treated the couple as the unit of the analysis, corresponding data from both partners of a couple were included in this analysis. Through an iterative process (c.f., Takács et al., 2015; Vargha et al., 2016) we identified the appropriate number of clusters. In the next analytic step, we deductively compared the clusters – representing subsamples of couples with specific stress and dyadic coping patterns in their personal projects – with respect to basic demographic characteristics. It was also tested whether the clusters differed in the outcome variables; partners’ positive emotions and sense of community as experienced in their stressful personal projects, as well as in relationship satisfaction of the partners. Cluster comparisons were performed using ANCOVA with Bonferroni *post hoc* test.

**Table 4:** Descriptive statistics, psychometric properties, and bivariate correlations for the variables.

					Pearson correlation coefficients											
					
		Range	*m*	*SD*	1	2	3	4	5	6	7	8	9	10	11	12
1	p-stress_M	1–7	4.12	1.26	*0,858*											
2	p-stress_F	1–7	4.11	1.19	0.407^∗∗^	*0,857*										
3	p-posDC_M	8–33	14.51	5.39	-0.120^∗^	-0.169^∗∗^	*0,875*									
4	p-posDC_F	8–31	14.30	5.40	-0,076	-0.199^∗∗^	0.456^∗∗^	*0,874*								
5	p-negDC_M	22–70	51.98	8.28	0.266^∗∗^	0.214^∗∗^	-0.346^∗∗^	-0.211^∗∗^	*0,830*							
6	p-negDC_F	26–70	52.05	8.25	0,098	0.285^∗∗^	-0.174^∗∗^	-0.409^∗∗^	0.498^∗∗^	*0,832*						
7	p-posem_M	1–7	4.46	1.51	-0.399^∗∗^	-0.292^∗∗^	0.316^∗∗^	0.137^∗^	-0.126^∗^	-0,013	*0,840*					
8	p-posem_F	1–7	4.51	1.42	-0.182^∗∗^	-0.447^∗∗^	0.175^∗∗^	0.271^∗∗^	-0,045	-0,099	0.399^∗∗^	*0,813*				
9	p-community_M	1–7	5.35	1.81	-0,002	-0.121^∗^	0.338^∗∗^	0,088	-0.179^∗∗^	-0,024	0.384^∗∗^	0.179^∗∗^	*0,814 ^1^*			
10	p-community_F	1–7	5.26	1.93	0,031	-0,015	0.216^∗∗^	0.315^∗∗^	-0,117	-0.127^∗^	0.159^∗∗^	0.433^∗∗^	0.374^∗∗^	*0,875 ^1^*		
11	RAS_M	16–35	30.65	4.17	-0.314^∗∗^	-0.289^∗∗^	0.392^∗∗^	0.389^∗∗^	-0.594^∗∗^	-0.420^∗∗^	0.195^∗∗^	0.198^∗∗^	0.159^∗∗^	0.173^∗∗^	*0,875*	
12	RAS_F	11–35	30.28	4.23	-0.216^∗∗^	-0.352^∗∗^	0.266^∗∗^	0.508^∗∗^	-0.414^∗∗^	-0.507^∗∗^	0.133^∗^	0.251^∗∗^	0,02	0.222^∗∗^	0.633^∗∗^	*0,859*


*p- prefix denotes personal project. Suffixes: M = male; F = female. posDC = positively balanced dyadic coping; negDC = negatively balanced dyadic coping; SFS = subjective financial status; posem = positive emotions; community = sense of community; RAS = Relationship Assessment Scale. Reliability estimates in the diagonal. ^*1*^Correlation coefficients instead of alphas. ^∗^*p* < 0.05, ^∗∗^*p* < 0.01. *N* = 267–270.*

### Preliminary Analyses

First we tested whether aggregated ratings of personal project experiences formed reliable subscales. Alpha coefficients (Table 4, diagonal) were appropriate in magnitude for all of the subscales; ranging from 0.813 (positive emotions in female respondents) to 0.875 (DCI, Negative Dyadic Coping in male respondents). For sense of community items we calculated inter item correlations as estimates of reliability (0.814 and 0.875 for male and female respondents, respectively). After initial data processing we ran descriptive statistics and bivariate correlations for the variables in the study (see Table 4). Inspection of bivariate correlations revealed that the association between the two genders on the same scores were of only medium to low effect size for all of the coping scales (*r* = 0.374, *p* < 0.001 to *r* = 0.498; *p* < 0.001) while RAS was strongly associated (*r* = 0.633, *p* < 0.001). The conceptually similar subscales of Positive Dyadic Coping and Project Positivity indicated medium associations (*r* = 0.316, *p* < 0.001 and *r* = 0.271, *p* < 0.001 for male and female partners, respectively) while there were low effect sizes between Negative Dyadic Coping and Project Stress (*r* = 0.266, *p* < 0.001 and *r* = 0.285, *p* < 0.001 for male and female partners, respectively).

The presence of partners with the same (vs. different) projects in the sample was considered to have a potentially confounding effect on results. Therefore, we also checked personal projects to identify partners with same projects; that is, we looked for cases where both partners named essentially identical projects (see Table 3 for examples). Two independent raters content-analyzed the individual project descriptions and decided whether descriptions referred to the same (vs. different) personal project between partners. The reliability of inter-rater decisions was assessed using intraclass correlation (two-way, consistency, average-measures ICC; Hallgren, 2012), yielding an ICC coefficient of 0.922 (95% CI = 0.902 – 0.939). Discrepancies were resolved by team discussion. In sum, 66 couples (24.6%) referenced the same projects. There were no statistically significant differences between couples with and without the same personal projects in terms of demographic variables (age, relationship status, relationship duration) nor several psychological characteristics (project stress, positive and negative dyadic coping, positive emotions in projects, as well as relationship satisfaction) after correcting the alpha level for multiple comparisons. We found only one significant difference; on average, partners with the same project had higher sense of community in their projects.^1^

### Cluster Analysis of the Stress and Dyadic Coping Variables

We conducted a series of hierarchical cluster analyses to determine the best-fitting cluster solution (c.f., Vargha et al., 2015) Cluster analysis was run on standardized scores of the initial variables (stress, positive and negative dyadic coping of both partners in their projects; in sum, six variables) via Ward method with squared Euclidean distances, which maximizes the difference between the groups and minimizes it between the clusters. Following the procedure described by Vargha et al. (2015), we compared 3–10 cluster solutions with regard to their adequacy. For each of these actual cluster solutions we examined the most important adequacy measures (explained variance, Point-bi-serial Correlation, Silhouette Coefficient, average cluster homogeneity; c.f., Vargha et al., 2016). Adequacy measures are presented in Table 5 for the cluster solutions with 3–10 clusters. We determined the final number of clusters based on inspection of the adequacy measures as well as preliminary interpretation of the potentially well fitting cluster solutions. As a result, we retained the six-cluster solution for further analysis because this solution maximized goodness of fit, explanatory power and interpretability. Comparison of the adequacy measures (see Table 5) shows that the *N* = 6 clusters solution is appropriate in several ways. First, EESS = 46.99%, which is adequate considering that we may expect it to increase after relocation (Vargha et al., 2016). Moreover, inspection of the change diagram of added EESS% showed that there is an elbow around solutions 6 and 7. This means that the addition of the seventh cluster adds proportionally less to this value than the previous solutions in terms of EESS%. Second, the point-bi-serial coefficient is well above the 0.3 threshold, while the Homogeneity Coefficients of the clusters are around 1.0. More importantly, the modified Xie-Beni index indicated a local maximum for this solution. This was interpreted to mean that the six-cluster solution was locally the most homogeneous compared to its neighbors. Preliminary interpretation of this solution also confirmed its viability. The next solution with a similar modified Xie-Beni index was the nine-cluster solution. However, retaining nine clusters would have led to challenges both in interpretation and because of the resulting low sample size of cluster memberships which may have produced non-significant associations in group comparison. Therefore, to maintain the robustness of results, we decided to apply the six-cluster solution for the analysis. Following the confirmation of the appropriate number of clusters, a relocation process was performed so that the individual cases matched their final cluster better. As a result, EESS% increased to 50.52%, and the modified Xie-Beni index remained above 0.3. Upon relocation, the individual cases were assigned to the clusters for further analysis. The proportion of each of the clusters in the examined sample can be regarded as fairly balanced, ranging from 10.37 to 24.07%.

**Table 5:** Adequacy indexes of cluster solutions 3–10.

Step	Cluster N	EESS %	Point biserial	XieBeni (mod)	Silhouette coefficient	HC mean
i = 260	10	58.43	0.291	0.127	0.430	0.861
i = 261	9	56.01	0.309	0.349	0.419	0.907
i = 262	8	53.31	0.309	-0.030	0.411	0.959
i = 263	7	50.39	0.333	0.278	0.432	1.015
i = 264	6	46.99	0.34	0.361	0.418	1.081
i = 265	5	43.00	0.331	0.281	0.405	1.157
i = 266	4	37.97	0.32	0.088	0.389	1.254
i = 267	3	30.04	0.312	-0.135	0.364	1.409
After relocation	6	50.52	0.358	0.333	0.473	1.009


*EESS = Explained Error Sum of Squares; Point biserial = point biserial correlation coefficient; XieBeni (mod) = modified Xie-Beni index; HC = Homogeneity of Cluster index.*

**FIGURE 1:**
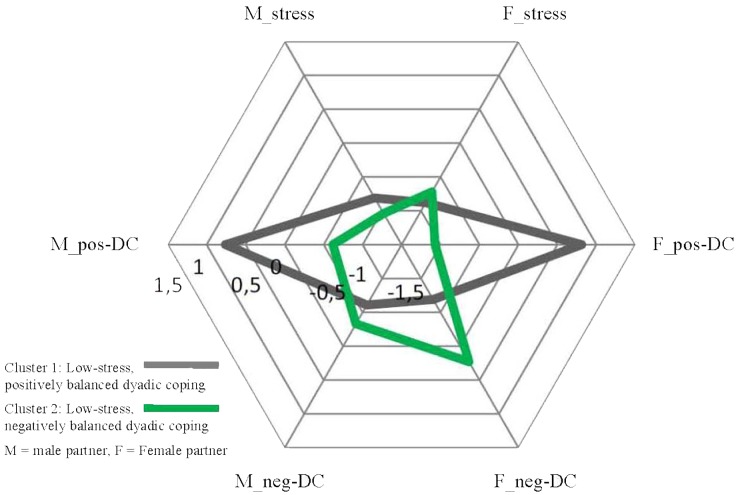
Low-stress couples: Cluster centers of Cluster 1 and 2, z-scores.

**FIGURE 2:**
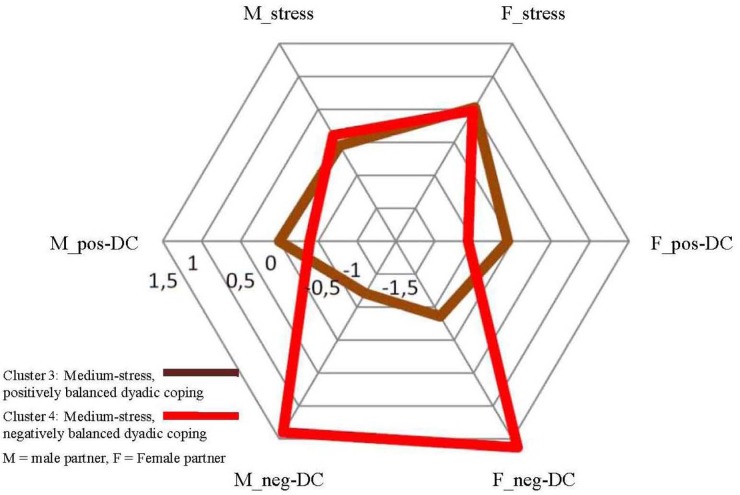
Medium-stress couples: Cluster centers of Cluster 3 and 4, z-scores.

**FIGURE 3:**
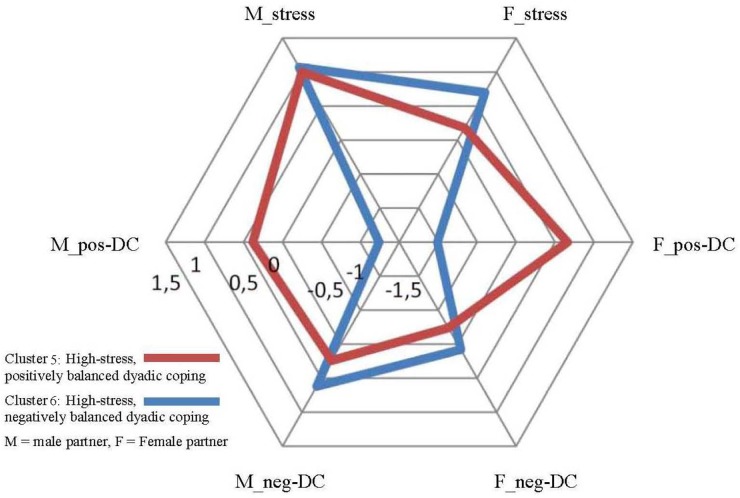
High-stress couples: Cluster centers of Cluster 5 and 6, z-scores.

We ran a series of ANOVAs to determine how the clusters as groups of couples were different in terms of the initial dimensions of stress and dyadic coping of the partners (see Table 6). Closer inspection of the cluster centers and their graphic representation (see Figure 1–3) indicated that cluster solutions could be grouped in a three- (lower, medium, and higher stress) -by-two (positively vs. negatively balanced dyadic coping pattern) array. Clusters 1 and 2 are characterized by a low level of experienced stress in the personal projects of the partners (z-scores between -1.04 and -0.72), Clusters 3 and 4 with a medium level of experienced stress (z-scores between -0.05 and 0.53) and Clusters 5 and 6 with a medium level of experienced stress (z-scores between 0.18 and 1.07). It is important to note that Clusters 3–4 and 5–6 differ primarily and significantly in terms of the scores of male partners while there are no significant differences between the female scores in these four clusters. Still, we propose the interpretation of ‘medium’ vs. ‘high’ level stress because partners in Clusters 5 and 6 struggle with significantly more total stress in their lives than couples in Clusters 3 and 4. However, in later interpretations it is important to keep in mind that the main difference can be traced back to the stress level of the male partners. Concerning the latter analytical element it was clear that in Clusters 1, 3, and 5 positive dyadic coping experiences outweigh negative dyadic coping experiences in the projects of both partners, while the opposite is true for the couples in Clusters 2, 4, and 6. In these clusters negative ways of dyadic coping are relatively more frequent than positive ways in both partners’ experiences. While this description is true for the balance between positive and negative dyadic coping, the actual levels of these strategies vary across patterns. The frequencies of positive and negative dyadic coping are not equal in the clusters; however, they do not follow a linear pattern either. For example, negative dyadic coping is extremely high in both partners in Cluster 4 together with only medium-level couple stress. In a similar way, a high level of positive dyadic coping is indicated in partners, especially in women, in Clusters 1 and 5; that is, in couples with low and high stress, while in Cluster 3 medium-level stress was accompanied with only a medium level of positive dyadic coping. With all these nuances in mind, in the subsequent analyses we refer to the clusters using the above-mentioned characteristics of low, medium, and high stress, as well as positively and negatively balanced dyadic coping experiences.

**Table 6:** Descriptive statistics and comparison of cluster centers.

Clusters of stress and dyadic coping strategies in stressful personal projects															

	Low stress posbalanced DC (*N* = 65)		Low stress negbalanced DC (*N* = 29)		Medium stress posbalanced DC (*N* = 55)		Medium stress negbalanced DC (*N* = 42)		High stress posbalanced DC (*N* = 51)		High stress negbalanced DC (*N* = 28)		*F*	*p*	η^2^
									
	*M*	*SD*	*M*	*SD*	*M*	*SD*	*M*	*SD*	*M*	*SD*	*M*	*SD*		
p-stress_M	-0.81^a^	0.68	-1.04^a^	0.73	-0.05^b^	0.53	0.11^b^	0.76	1.00^c^	0.58	1.07^c^	0.62	75.31	<0.001	0.59
p-stress_F	-0.88^a^	0.71	-0.72^a^	0.74	0.53^b^	0.77	0.48^b^	0.75	0.18^b^	0.96	0.70^b^	0.72	34.27	<0.001	0.39
p-negDC_M	-0.61^a^	0.52	-0.33^a^	0.56	-0.71^a^	0.41	1.40^b^	0.89	0.24^c^	0.78	0.62^c^	0.87	67.63	<0.001	0.56
p-posDC_M	0.76^a^	0.86	-0.62^b^	0.97c	0.00 ^bc^	0.64	-0.39^ac^	0.76	0.38^d^	0.82	-1.24^d^	0.64	70.83	<0.001	0.57
p-negDC_F	-0.69^a^	0.52	0.23^b^	0.96	-0.36^ac^	0.62	1.63^d^	0.72	-0.24^c^	0.67	0.08^bc^	0.52	33.70	<0.001	0.39
p-posDC_F	0.81^a^	0.72	-1.07^b^	0.79	-0.06^c^	0.57	-0.57^d^	0.74	0.66^a^	0.58	-1.01^bd^	0.86	58.46	<0.001	0.53


*p- prefix denotes personal project. Suffixes: M = male; F = female. posDC = positive dyadic coping strategies; negDC = negative dyadic coping strategies; posbalanced DC = positively balanced dyadic coping; negbalanced DC = negatively balanced dyadic coping. Means without the same letters (in superscript) in a row are different at *p* < 0.05 level using Bonferroni adjustment in *post hoc test.**

### Comparison of the Clusters

Furthermore, one-way ANOVAs were conducted to detect differences between the six clusters regarding the sociodemographic characteristics of the partners. There was no significant difference in the partners’ age [*F*(5, 264) = 0.827, *p* = 0.532; *F*(5, 264) = 0.906, *p* = 0.478 for male and female partners, respectively), length of relationship [*F*(5, 206) = 0.317, *p* = 0.902; *F*(5, 201) = 0.368, *p* = 0.870] and number of underage children [*F*(5, 134) = 1.170, *p* = 0.327; *F*(5, 134) = 0.679, *p* = 0.640]. In contrast, the analysis showed significant differences for subjective financial status regarding both genders [*F*(5, 261) = 2.864, *p* = 0.015; *F*(5, 263) = 3.526, *p* = 0.004]. Bonferroni *post hoc* analysis showed that in couples with a low level of experienced stress and positive coping both spouses reported the highest subjective financial status (Cluster 1) while, unexpectedly, in couples with a medium level of experienced stress and negative coping (Cluster 4) the lowest subjective financial status level was reported (*p* = 0.019 and *p* = 0.004). There was no significant association between cluster membership and relationship status (marriage vs. cohabitation) [chi-square = 1.29 (5), *p* = 0.936] and between cluster membership and education either [chi-square = 14.49 (10), *p* = 0.152 and chi-square = 14.35 (10), *p* = 0.158, for male and female partners, respectively].

**FIGURE 4:**
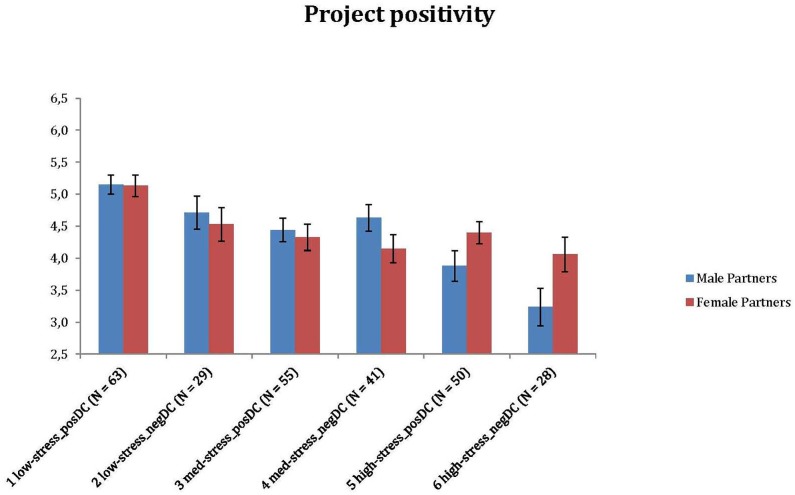
Differences of clusters: positive emotions in personal project.

**FIGURE 5:**
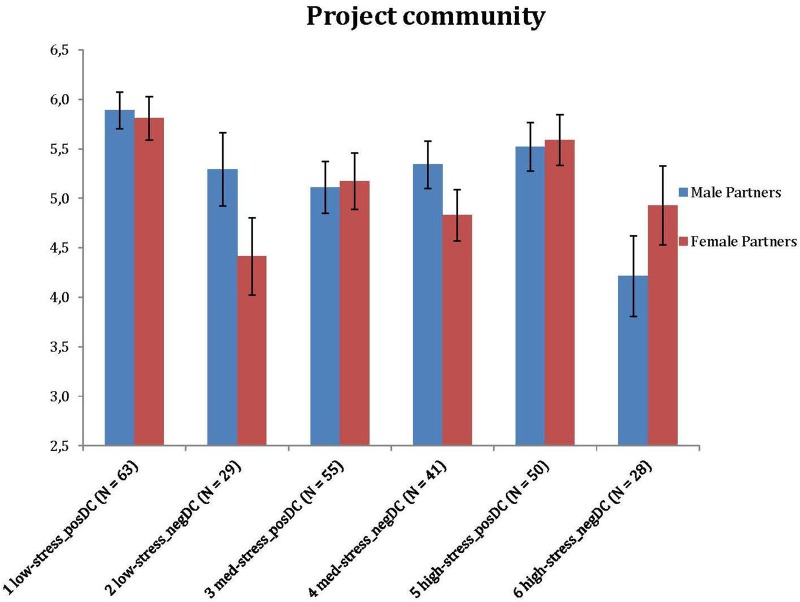
Differences of clusters: sense of community in personal project.

**FIGURE 6:**
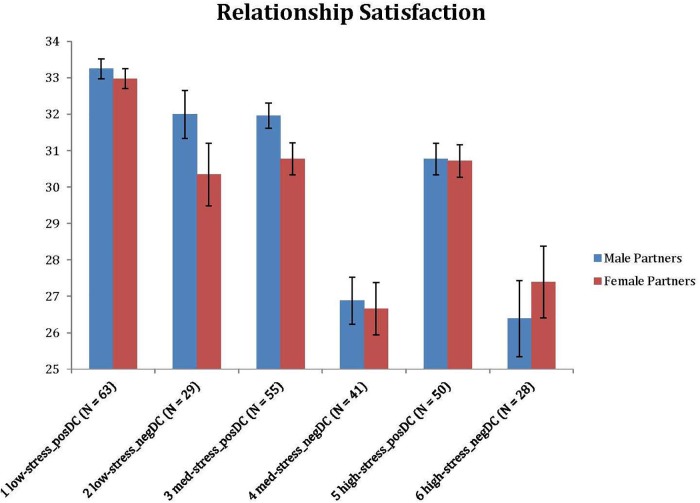
Differences of clusters: relationship satisfaction.

In the next step, we tested whether couples in the six clusters were different across a series of outcome measures, such as the partners’ positive emotions and sense of community experienced in their own projects, as well as their relationship satisfaction (see Figure 4–6). Since the preliminary analyses partly showed the significant associations of the clusters with sociodemographic characteristics, we performed subsequent group comparisons using a series of ANCOVAs, where the main effects were controlled for the subjective financial status scores of both partners. We assessed group differences using *post hoc* tests with Bonferroni adjustment. There were significant differences for both spouses for project positivity [*F*(5, 258) = 8.608, *p* < 0.001, η^2^ = 0.143; *F*(5, 258) = 2.815, *p* = 0.017, η^2^ = 0.052], project community [*F*(5, 258) = 3.911, *p* = 0.002, η^2^ = 0.070; *F*(5, 258) = 2.861, *p* = 0.016, η^2^ = 0.053] and relationship satisfaction [*F*(5, 256) = 25.467, *p* < 0.001, η^2^ = 0.332; *F*(5, 257) = 15.192, *p* < 0.001, η^2^ = 0.228]. Means and standard deviations for the clusters with regard to the partners’ project positivity, project community and RAS scores are presented in Table 7. Further investigation of the clusters as subgroups indicates the general trend that couples with lower (vs. higher) stress along with more positively (vs. negatively) balanced dyadic coping appear to experience better functioning in projects and higher relationship satisfaction. However, there are notable exceptions – for example, couples with lower stress but rather negatively balanced dyadic coping exhibit fairly high satisfaction but low positive emotions and sense of community in their projects.

**Table 7:** Comparisons of clusters across outcome measures.

Clusters of stress and dyadic coping strategies in stressful personal projects															

	Low stress posbalanced DC (*N* = 65)		Low stress negbalanced DC (*N* = 29)		Medium stress posbalanced DC (*N* = 55)		Medium stress negbalanced DC (*N* = 42)		High stress posbalanced DC (*N* = 51)		High stress negbalanced DC (*N* = 28)		*F*	*p*	η^2^
									
	*M*	*SD*	*M*	*SD*	*M*	*SD*	*M*	*SD*	*M*	*SD*	*M*	*SD*			
**ANOVA^1^**															
SFS_M	6.70^a^	1.75	6.38^ab^	1.50	5.93^ab^	1.67	5.51^b^	2.11	5.76^ab^	1.87	6.25^ab^	1.94	2.86	0.02	0.05
SFS_F	6.66^a^	1.47	6.07^ab^	1.58	6.05^ab^	1.68	5.38^b^	2.00	5.72^ab^	1.99	5.54^ab^	1.90	3.53	<0.001	0.06
ANCO VA^2^															
p-posem _M	5.15^a^	1.17	4.71^ab^	1.40	4.44^ab^	1.36	4.63^ab^	1.33	3.88^bc^	1.69	3.24^c^	1.54	8.61	<0.001	0.14
p-posem _F	5.13^a^	1.35	4.53^ab^	1.41	4.33^ab^	1.52	4.15^ab^	1.41	4.40^ab^	1.24	4.06^b^	1.43	2.82	0.02	0.05
p-community _M	5.89^a^	1.49	5.29^ab^	1.98	5.11^ab^	1.94	5.34^ab^	1.53	5.52^a^	1.73	4.21^b^	2.14	3.91	<0.001	0.07
p-community_F	5.81^a^	1.72	4.41^b^	2.09	5.17^ab^	2.11	4.83^ab^	1.65	5.59^ab^	1.83	4.93^ab^	2.10	2.86	0.02	0.05
RAS_M	33.25^a^	2.17	32.00^ab^	3.54	31.96^ab^	2.57	26.88^c^	4.13	30.77^b^	2.99	26.39^c,3^	5.53	25.47	<0.001	0.33
RAS_F	32.98^a^	2.12	30.34^ab^	4.61	30.78^a^	3.25	26.66^bc^	4.59	30.72^a^	3.18	27.39^b^	5.22	15.19	<0.001	0.23


*p- prefix denotes personal project. Suffixes: M = male; F = female. posbalanced DC = positively balanced dyadic coping; negbalanced DC = negatively balanced dyadic coping; SFS = subjective financial status; posem = positive emotions; community = sense of community; RAS = Relationship Assessment Scale. ^*1*^*F*-values are for ANOVA. ^*2*^*F*-values are for ANCOVA, controlled for subjective financial status. ^*3*^*N* = 48. Means and standard deviations of the subsamples are unadjusted values, where means without the same letters (in superscript) in a row are different at *p* < 0.05 level using Bonferroni adjustment in the *post hoc test*.*

### Additional Computations

In a subsequent series of analyses, we tested whether the results with the clusters were dependent on the proportion of couples with the same (vs. different) projects. First, we compared the frequency of couples with the same project in the six clusters. Chi-square statistics indicated that the differences in distribution were statistically not significant [χ^2^ = 5.15 (5), *p* = 0.398]. We also ran the above-described ANCOVAs with an additional covariate that coded shared (vs. not shared) projects in couples. Results indicated only minor differences in comparison to the above-presented data.^1^

## Discussion

In the present study, we examined the personal project-related stress and dyadic coping experiences of adult Hungarian couples who were living together in a committed long-term relationship. To the best of our knowledge, this is the first attempt to study these relationship processes via personal project assessment. Using a pattern-oriented approach to dyadic data analysis, we were able to extract six meaningfully different stress and dyadic coping clusters that captured distinct patterns of couples’ relationship processes connected to the personal project pursuit of the partners. Furthermore, we demonstrated that these patterns represented the non-linear and multidimensional nature of stress and dyadic coping in couples. We have argued that, through assessing dyadic coping processes in personal projects, the dyadic regulation of everyday pursuits can be studied in an ecologically valid way. Moreover, this approach may extend and add to the concept of dyadic coping as formulated in STM. Consequently, clusters of stress and dyadic coping represent different types of dyadic regulation processes as they are embedded in the everyday lives of couples. In the discussion below, we focus primarily on the potential implications and consequences of these relationship-level regulatory patterns.

### Relationship-Level Patterns of Stress and Dyadic Coping

Through cluster analysis we identified six clusters with specific patterns of stress and dyadic coping experiences in the personal projects of the partners, where clusters were subsamples of couples. The clusters could be roughly characterized by two features: the level of stress experienced by the partners (lower, medium and higher stress) coupled with a positively vs. negatively balanced dyadic coping style. These patterns were mainly independent of sociodemographic characteristics, such as age, education, and length of relationship; this points to their potential generalizability as everyday regulatory strategies. However, we found significant associations with the subjective appraisals of financial status of the respondents. Closer inspection of these patterns generates a series of conclusions about the relationship-level regulation of everyday goals.

First, clusters represent non-linear, relationship-level associations between stress and dyadic coping experiences of couples. In bivariate associations, experienced stress was clearly directly correlated to negative (and in inverse correlation with positive) dyadic strategies within individual responses and partly also between partners (although effect sizes were typically in the low-to-medium range). Moreover, correlations between negative and positive dyadic coping strategies were also inverse and in the medium range. However, clusters represented systematic exceptions to these findings; at every level of stress, dyadic coping strategies showed both combinations of positively and negatively balanced coping patterns. Accordingly, we were able to identify “out of the rule” subgroups of couples. For example, in Cluster 5, a high level of stress appeared together with the relative dominance of positive dyadic coping in partners. Furthermore, in Cluster 4, negative dyadic coping was at its highest in both partners from among all clusters, in spite of only a medium level of stress.

Second, the detected patterns showed considerable symmetry; that is, relatively high “agreement” between partners with respect to the measured characteristics. Since the projects that were assessed in this procedure were individually chosen and personally important, this high agreement may represent deeper interdependence between the partners’ self-regulation processes. Interdependence can appear in at least two ways; the projects may be the same or closely interconnected (e.g., quit smoking because of the birth of a baby; couple No. 63 in Table 3) and the way the partners pursue them may be interconnected too (e.g., when partners mutually support each other’s important – but different – pursuits; Kaplan and Maddux, 2002; Kuster et al., 2017). Both possibilities are in line with previous theorizing about goal interdependence in close relationships (Fitzsimons and Finkel, 2015).

Finally, our results confirm that a pattern oriented approach to dyadic data is an appropriate way to explore complex interaction patterns in a series of characteristics, in our case, stress and dyadic coping appraisals of partners. Studies on pattern-oriented approach assert that the strength of a pattern-oriented approach lies in its potential to detect non-linear, “outside the general rule” variations in the complex associations of relationship constructs (e.g., Gagliardi et al., 2013; Cao et al., 2015; Czikmantori et al., 2018), while preserving the holistic view of a couple’s relationship. Non-linearity was not only present in the emergent clusters of stress and dyadic coping but also in their associations with outcome characteristics.

### Associations Between Stress and Dyadic Coping Patterns With Outcomes

We assumed that specific patterns of stress and dyadic coping experiences might be associated with the partners’ functioning in their personal projects (positive emotions and sense of community) and with their relationship satisfaction. Confirming this assumption in the deductive analytic step of the study, we found significant differences between the clusters with regard to these outcomes. On the whole, higher experienced stress in projects was only associated with adverse outcomes when it was also associated with the higher prevalence of negative and lower prevalence of positive dyadic coping strategies. This was primarily true for lower sense of community in the personal project and lower general relationship satisfaction. In contrast, medium and high experienced stress was buffered against adverse effects by a positively balanced coping pattern in the projects. Associations of positive emotional experiences deviated from this trend. Couples that experienced lower stress in their projects felt generally more positive emotions in their projects than higher stressed couples, independent of the quality of their dyadic coping experiences. Below we give detailed implications of these results. We also provide further considerations on additional findings in Appendix 1.

#### Positive Emotions in Personal Project

In our study, positive emotions referred to primarily individual experiences of the partners in their personal projects. This way, the direct link of higher perceived stress to diminishing positive emotions may indicate a specific risk to health and well-being of the partners. According to an earlier longitudinal study, greater decreases in positive affect in response to daily stressors represent a risk of higher mortality (Mroczek et al., 2013). However, recent research has also found that the adaptive regulation of emotional states in the individual is connected to more positive dyadic coping of the partners (Rusu et al., 2018) and that positive emotions play a crucial role in successful coping processes (c.f., Folkman, 2008). Therefore, later research may address how and to what extent the appropriate use of positive dyadic strategies can still help couples to preserve positive emotional experiences even during the accomplishment of stressful personal projects.

#### Sense of Community in Personal Project

For both genders, complex patterns of stress and dyadic coping strategies were in a significant non-linear association with sense of community, a primarily relational experience of the partners in their personal projects. Male partners experienced the strongest sense of community both when dealing with low- and high-stress projects, paired with a positive dyadic coping pattern in their relationships. Female partners’ sense of community was highest when low-stress projects were paired with positive coping patterns in the couple. Similarly, with negatively balanced dyadic coping strategies in relationship-level regulation of personal projects, male and female partners showed divergent vulnerabilities to diminished sense of community. Women were especially sensitive to low-level stress and negative dyadic coping (Cluster 2) in their sense of community, while men felt similarly when the partners were highly stressed in their projects, paired with negative dyadic coping (Cluster 6).

The protection of sense of community in personal projects – even in the face of high stress and lower actual positive emotions – may be beneficial for the short- and long-term functioning of relationships in many ways. The mutual pursuit of goals has been found to be associated with better relationship functioning, higher support and better goal progress (Kaplan and Maddux, 2002; Avivi et al., 2009) and the mutual sharing of future selves may support enjoyment in cooperation and personal well-being (Schindler et al., 2010). On the other hand, lack of goal similarity and joint goal planning may be indicators of the long-term risk of relationship dissolution (Arránz Becker, 2013; Gere et al., 2016), presumably because joint goal pursuits help partners to better align themselves with each other, and to involve each other in important strivings and the related decisions. Finally, emotional and practical coordination of important personal pursuits may extend and reinforce the partners’ communal orientation which in turn may support further dyadic coping efforts (Randall et al., 2013; Koranyi et al., 2017).

#### Relationship Satisfaction

The interaction between stress level and the quality of dyadic coping strategies applied by the couple to cope with this stress was especially evident when we focused on outcomes in the context of the relationship in general, that is, in the case of relationship satisfaction. Clusters with a positive dyadic coping style in personal projects – even when paired with medium or high stress – were associated with higher relationship satisfaction than clusters with negative balance in dyadic coping. These associations had considerable effect sizes, and clearly showed that positively tuned dyadic coping strategies in personal projects may mitigate the adverse effect of heightened stress. Moreover, such findings are comparable to those of previous research about the stress-buffering effect of positive dyadic coping (Merz et al., 2014; Falconier et al., 2015b; Breitenstein et al., 2018; Hilpert et al., 2018). It is important to note, however, that project-related stress cannot be identified as purely external or internal; therefore dyadic coping in personal projects can be conceptualized as much as *managing* stress as just *buffering against* stress spillover. Consequently, higher relationship satisfaction may be regarded as a resource for stress management of the couple. The significance of high relationship satisfaction for relational self-regulation is highlighted by the fact that it may promote the effective goal pursuit of partners (Hofmann et al., 2015; Cappuzzello and Gere, 2018). Moreover, couples who were more satisfied with their relationship were more likely to choose to resolve stressful situations in a cooperative way (Bodenmann and Cina, 2000; Koranyi et al., 2017). These studies point to the possibility of a bidirectional association between stress, dyadic coping and relationship satisfaction. Research on relationship-level processes of personal project pursuit may add further insights into these phenomena.

### Limitations

In interpreting the results of the current paper, one should bear in mind certain limitations. First, the cross-sectional research design does not allow for causal explanations of the results. Second, due to our reliance on self-reported responses solely, the validity of answers might be affected. Third, since the examined sample consisted of Hungarian respondents exclusively, future research can aim at investigating the cross-cultural generalizability of stress and dyadic coping patterns. Fourth, we assessed only one personal project for each respondent – later studies may use a more extended measurement of personal projects and the experiences associated with them. Fifth, we did not assess the other partner’s real experiences concerning the personal project of one partner, but relied only on his or her perceptions. This leaves open the possibility that perceptions on the other partner’s stress, positive emotions, or even dyadic coping behaviors were biased to a certain extent. Sixth, our emergent clusters represent complex interactions of multiple characteristics in an explorative, heuristic way. Later research should test these associations with more theoretically driven, targeted moderation analyses, for example in form of two- or three-way interaction analyses between gender, stress and dyadic coping. Finally, in order to make the cluster solutions easier to interpret, we focused on aggregated positive and negative dyadic coping scores only. A finer-grained analysis that differentiates between self and partner aspects and several forms of dyadic coping may strengthen the scientific power of the approach presented here.

## Conclusion and Future Directions

Our research documents the protective role of positively balanced dyadic coping strategies in the pursuit of everyday personal projects of people living in committed relationships. Dyadic coping processes proved to be important elements of the systemic, relationship-level regulation of personal projects in couples and when positively balanced, they predicted higher quality of relationship functioning, even when projects were rather stressful. These results have many implications for methodology, theory and the potential applications of dyadic coping research.

First, the results confirm previous notions that dyadic coping can be studied in a psychometrically reliable and ecologically valid way using personal projects as units (c.f., Martos et al., 2019). Through the dyadic coping-focused assessment of personal projects we can tackle everyday relationship-level experiences and the related regulatory strategies of couples. Later studies could use personal project assessment in the study of dyadic coping with specific life circumstances, like chronic illness or financial strain (c.f., Meier et al., 2011; Traa et al., 2015).

Second, on a more theoretical level the results also demonstrate that the pursuit of personally relevant projects is deeply connected to the interpersonal reality of a couple’s relationship (c.f., Fitzsimons et al., 2015) while, by definition, they reflect personal and contextual features at the same time (Little, 2006). More or less consciously, people may face a considerable amount of stress when striving toward important personal goals. In other instances, life challenges arise, and projects are used to handle the resulting stress. Important relationships may play a role in both processes. Moreover, later research may address how overarching pursuits, like personal projects are modified through the actual dyadic coping processes: the activated coping goals (c.f., Bodenmann, 1995; Bodenmann et al., 2016) and the resulting experiences of positive emotions and sense of community. In sum, we may assume that a series of complex intra- and interpersonal transactions exist involving relational features – such as dyadic coping strategies – and contextual-environmental conditions and challenges. The dynamics and details of these transactional processes should be a target of later studies: understanding the former may deepen our understanding of relationship functioning as they are embedded in everyday life situations.

Finally, the results of the pattern-oriented approach presented here have implications for praxis. Identification of high-risk relationship patterns in couples may increase the sensitivity of practitioners to special configurations of vulnerability. Our results also confirm the importance of specific situations, such as financial strain, a frequent latent stressor for many couples (c.f., Martos et al., 2016). In these situations, application of STM-based training programs like Couples Coping Enhancement Training (CCET, Bodenmann and Shantinath, 2004) and TOGETHER (Falconier, 2015) may help couples to improve their individual and dyadic coping strategies. In our sample, partners who had highly stressful personal projects but applied positive dyadic coping strategies demonstrated the opportunity of fulfilling bonds and shared sense of community, even when they faced challenges in their life pursuits.

## Data Availability

The raw database for this study that supports the conclusions of this manuscript will be made available by the authors, without undue reservation, to any qualified researcher.

## Author Contributions

TM and VS designed the study and carried out the research. TM and OF conducted data analysis. TM and HG conducted content analysis of personal projects. TM, VS, and OF wrote sections of the manuscript. HG and MN did the literature review and revised the manuscript. All authors contributed to the final interpretation of findings and read and approved this final version.

## Conflict of Interest Statement

The authors declare that the research was conducted in the absence of any commercial or financial relationships that could be construed as a potential conflict of interest.

## Footnotes

1 Results are available on request from the corresponding author.

## Appendix 1: Discussion of Additional Findings

### The Role of Perceived Financial Status in Stress and Coping Patterns

One contextual feature that has received attention in recent studies related to relationship functioning and dyadic coping is the financial situation of couples. Research findings have confirmed that financial distress significantly increases the risk of negative impacts on relationship satisfaction and that this risk is modulated by the way couples manage financial challenges in their daily interactions (Archuleta et al., 2011; Barton et al., 2015). In fact, financial strain may lead to relationship dissatisfaction and dissolution when coupled with severe disagreement, while financial disagreements are among the most severe types of disagreement in relationships (Dew et al., 2012; Dew and Dakin, 2011).

As a corollary finding that fits this trend, we have documented how the stress and dyadic coping clusters were significantly different with regard to the level of subjective financial status of both partners. Interestingly, the lowest levels of perceived financial status were found in Cluster 4 in which stress was only at a medium level but negative dyadic coping was highest in both partners. Although general patterns of associations remained significant after controlling for financial status variables, later studies may include and scrutinize measures of financial status as well.

### The Role of Shared Projects

We also tested whether our results were affected by the presence of certain couples’ shared projects in the sample. Roughly a quarter of the couples specified the same project as most stressful. However, inclusion of this distinction in the analyses did not change the results considerably; the proportion of couples with shared projects was quite stable across the clusters, while associations of clusters with outcomes remained largely unaffected by this variable as well. While null findings have to be interpreted cautiously, we conclude that our results show the relative importance of general regulation processes over the specific content of personal projects. Regardless of their shared vs. non-shared content, individual personal projects are jointly regulated by the couples, and the associated stress and dyadic coping experiences seem to represent important aspects of relationship functioning. Later investigations may strengthen these notions or confirm the potentially distinctive role of shared personal project pursuits in relationship functioning.
